# Supplementary material for: Validation of the Malawi Developmental Assessment Tool for children in the Dominican Republic: Preliminary results
Source: PLoS One. 2019 Aug 15;14(8):e0221162. doi: 10.1371/journal.pone.0221162 (PMC6695133; doi:10.1371/journal.pone.0221162)
Supplement: S1 File — (PDF) [file pone.0221162.s001.pdf]

Codebook

Notes

**Output Created**		**02-AUG-2019 13:41:03**
**Comments**		
**Input**	**Data**	/Users/lauvsanchezv/Google Drive/Tulane University/Estudio de viabilidad del MDAT/PLOS ONE/Sánchez-Vincitore, Schaettle, & Castro 2019/Database.sav
	**Active Dataset**	**DataSet1**
	**Filter**	**<none>**
	**Weight**	**<none>**
	**Split File**	**<none>**
	N of Rows in Working Data File	**42**

Notes

**Syntax**	CODEBOOK ParticipantCode [s] Age [o] Sex [n] A1 [n] A2 [n] A3 [n] A4 [n] A5 [n] A6 [n] A7 [n] A8 [n] A9 [n] A10 [n] A11 [n] A12 [n] A13 [n] A14 [n] A15 [n] A16 [n] A17 [n] A18 [n] A19 [n] A20 [n] A21 [n] A22 [n] A23 [n] A24 [n] A25 [n] A26 [n] A27 [n] A28 [n] A29 [n] A30 [n] A31 [n] A32 [n] A33 [n] A34 [n] B1 [n] B2 [n] B3 [n] B4 [n] B5 [n] B6 [n] B7 [n] B8 [n] B9 [n] B10 [n] B11 [n] B12 [n] B13 [n] B14 [n] B15 [n] B16 [n] B17 [n] B18 [n] B19 [n] B20 [n] B21 [n] B22 [n] B23 [n] B24 [n] B25 [n] B26 [n]<br>B27 [n] B28 [n] B29 [n] B30 [n] B31 [n] B32 [n] B33 [n] B34 [n] C1 [n] C2 [n] C3 [n] C4 [n] C5 [n] C6 [n] C7 [n] C8 [n] C9 [n] C10 [n] C11 [n] C12 [n] C13 [n] C14 [n] C15 [n] C16 [n] C17 [n] C18 [n] C19 [n] C20 [n] C21 [n] C22 [n] C23 [n] C24 [n] C25 [n]<br>C26 [n] C27 [n] C28 [n] C29 [n] C30 [n] C31 [n] C32 [n] C33 [n] C34 [n] D1 [n] D2 [n] D3 [n] D4 [n] D5 [n] D6 [n] D7 [n] D8 [n] D9 [n] D10 [n] D11 [n] D12 [n] D13 [n] D14 [n] D15 [n] D16 [n] D17 [n] D18 [n] D19 [n] D20 [n] D21 [n] D22 [n] D23 [n] D24 [n]<br>D25 [n] D26 [n] D27 [n] D28 [n] D29 [n] D30 [n] D31 [n] D32 [n] D33 [n] D34 [n] E1 [n] E2 [n] BooksYesNo [n] E3 [n] E4 [n] E5 [n] E6 [n] E7 [n] E8 [n] E9 [n] E10.1 [n] E10.2 [n] E10.3 [n] E10.4 [n] E10.5 [n] E10.6 [n] E10.7 [n] E10.8 [n] E10.9 [n] E10.10<br>[n] E10.11 [n] E10.12 [n] E10.13 [n] E10.14 [n] E10.15 [n] E10.16 [n] E11 [n] E12 [n] E13 [n] TotalA [n] TotalB [n] TotalC [n] TotalD [n]<br>/VARINFO LABEL TYPE VALUELABELS<br>/OPTIONS VARORDER=VARLIST SORT=ASCENDING MAXCATS=200<br>/STATISTICS NONE.

Notes

Resources	Processor Time	00:00:00.11
	Elapsed Time	00:00:01.00

ParticipantCode

		Value
Standard Attributes	Label	Code
	Type	Numeric

Age

		Value
Standard Attributes	Label	Age in months
	Type	Numeric

Sex

		Value
Standard Attributes	Label	Sex
	Type	String

A1

		Value
Standard Attributes	Label	Social1
	Type	Numeric

A2

		Value
Standard Attributes	Label	Social2
	Type	Numeric

A3

		Value
Standard Attributes	Label	Social3
	Type	Numeric

A4

		Value
Standard Attributes	Label	Social4
	Type	Numeric

A5

		Value
Standard Attributes	Label	Social5
	Type	Numeric

A6

		Value
Standard Attributes	Label	Social6
	Type	Numeric

A7

		Value
Standard Attributes	Label	Social7
	Type	Numeric

A8

		Value
Standard Attributes	Label	Social8
	Type	Numeric

A9

		Value
Standard Attributes	Label	Social9
	Type	Numeric

A10

		Value
Standard Attributes	Label	Social10
	Type	Numeric

A11

		Value
Standard Attributes	Label	Social11
	Type	Numeric

A12

		Value
Standard Attributes	Label	Social12
	Type	Numeric

A13

		Value
Standard Attributes	Label	Social13
	Type	Numeric

A14

		Value
Standard Attributes	Label	Social14
	Type	Numeric

A15

		Value
Standard Attributes	Label	Social15
	Type	Numeric

A16

		Value
Standard Attributes	Label	Social16
	Type	Numeric

A17

		Value
**Standard Attributes**	**Label**	Social17
	**Type**	**Numeric**

A18

		Value
**Standard Attributes**	**Label**	Social18
	**Type**	**Numeric**

A19

		Value
**Standard Attributes**	**Label**	Social19
	**Type**	**Numeric**

A20

		Value
**Standard Attributes**	**Label**	Social20
	**Type**	**Numeric**

A21

		Value
**Standard Attributes**	**Label**	Social21
	**Type**	**Numeric**

A22

		Value
**Standard Attributes**	**Label**	Social22
	**Type**	**Numeric**

A23

		Value
**Standard Attributes**	**Label**	Social23
	**Type**	**Numeric**

A24

		Value
**Standard Attributes**	**Label**	Social24
	**Type**	**Numeric**

A25

		Value
**Standard Attributes**	**Label**	Social25
	**Type**	**Numeric**

A26

		Value
**Standard Attributes**	**Label**	Social26
	**Type**	**Numeric**

A27

		Value
Standard Attributes	Label	Social27
	Type	**Numeric**

A28

		Value
Standard Attributes	Label	Social28
	Type	**Numeric**

A29

		Value
Standard Attributes	Label	Social29
	Type	**Numeric**

A30

		Value
Standard Attributes	Label	Social30
	Type	**Numeric**

A31

		Value
Standard Attributes	Label	Social31
	Type	**Numeric**

A32

		Value
Standard Attributes	Label	Social32
	Type	**Numeric**

A33

		Value
Standard Attributes	Label	Social33
	Type	**Numeric**

A34

		Value
Standard Attributes	Label	Social34
	Type	**Numeric**

B1

		Value
Standard Attributes	Label	FineMotor1
	Type	**Numeric**

B2

		Value
Standard Attributes	Label	FineMotor2
	Type	**Numeric**

B3

		Value
Standard Attributes	Label	FineMotor3
	Type	Numeric

B4

		Value
Standard Attributes	Label	FineMotor4
	Type	Numeric

B5

		Value
Standard Attributes	Label	FineMotor5
	Type	Numeric

B6

		Value
Standard Attributes	Label	FineMotor6
	Type	Numeric

B7

		Value
Standard Attributes	Label	FineMotor7
	Type	Numeric

B8

		Value
Standard Attributes	Label	FineMotor8
	Type	Numeric

B9

		Value
Standard Attributes	Label	FineMotor9
	Type	Numeric

B10

		Value
Standard Attributes	Label	FineMotor10
	Type	Numeric

B11

		Value
Standard Attributes	Label	FineMotor11
	Type	Numeric

B12

		Value
Standard Attributes	Label	FineMotor12
	Type	Numeric

B13

		Value
**Standard Attributes**	**Label**	FineMotor13
	**Type**	**Numeric**

B14

		Value
**Standard Attributes**	**Label**	FineMotor14
	**Type**	**Numeric**

B15

		Value
**Standard Attributes**	**Label**	FineMotor15
	**Type**	**Numeric**

B16

		Value
**Standard Attributes**	**Label**	FineMotor16
	**Type**	**Numeric**

B17

		Value
**Standard Attributes**	**Label**	FineMotor17
	**Type**	**Numeric**

B18

		Value
**Standard Attributes**	**Label**	FineMotor18
	**Type**	**Numeric**

B19

		Value
**Standard Attributes**	**Label**	FineMotor19
	**Type**	**Numeric**

B20

		Value
**Standard Attributes**	**Label**	FineMotor20
	**Type**	**Numeric**

B21

		Value
**Standard Attributes**	**Label**	FineMotor21
	**Type**	**Numeric**

B22

		Value
**Standard Attributes**	**Label**	FineMotor22
	**Type**	**Numeric**

B23

		Value
**Standard Attributes**	**Label**	FineMotor23
	**Type**	**Numeric**

B24

		Value
**Standard Attributes**	**Label**	FineMotor24
	**Type**	**Numeric**

B25

		Value
**Standard Attributes**	**Label**	FineMotor25
	**Type**	**Numeric**

B26

		Value
**Standard Attributes**	**Label**	FineMotor26
	**Type**	**Numeric**

B27

		Value
**Standard Attributes**	**Label**	FineMotor27
	**Type**	**Numeric**

B28

		Value
**Standard Attributes**	**Label**	FineMotor28
	**Type**	**Numeric**

B29

		Value
**Standard Attributes**	**Label**	FineMotor29
	**Type**	**Numeric**

B30

		Value
**Standard Attributes**	**Label**	FineMotor30
	**Type**	**Numeric**

B31

		Value
**Standard Attributes**	**Label**	FineMotor31
	**Type**	**Numeric**

B32

		Value
**Standard Attributes**	**Label**	FineMotor32
	**Type**	**Numeric**

B33

		Value
**Standard Attributes**	**Label**	FineMotor33
	**Type**	**Numeric**

B34

		Value
**Standard Attributes**	**Label**	FineMotor34
	**Type**	**Numeric**

C1

		Value
**Standard Attributes**	**Label**	Language1
	**Type**	**Numeric**

C2

		Value
**Standard Attributes**	**Label**	Language2
	**Type**	**Numeric**

C3

		Value
**Standard Attributes**	**Label**	Language3
	**Type**	**Numeric**

C4

		Value
**Standard Attributes**	**Label**	Language4
	**Type**	**Numeric**

C5

		Value
**Standard Attributes**	**Label**	Language5
	**Type**	**Numeric**

C6

		Value
**Standard Attributes**	**Label**	Language6
	**Type**	**Numeric**

C7

		Value
**Standard Attributes**	**Label**	Language7
	**Type**	**Numeric**

C8

		Value
**Standard Attributes**	**Label**	Language8
	**Type**	**Numeric**

C9

		Value
Standard Attributes	Label	Language9
	Type	Numeric

C10

		Value
Standard Attributes	Label	Language10
	Type	Numeric

C11

		Value
Standard Attributes	Label	Language11
	Type	Numeric

C12

		Value
Standard Attributes	Label	Language12
	Type	Numeric

C13

		Value
Standard Attributes	Label	Language13
	Type	Numeric

C14

		Value
Standard Attributes	Label	Language14
	Type	Numeric

C15

		Value
Standard Attributes	Label	Language15
	Type	Numeric

C16

		Value
Standard Attributes	Label	Language16
	Type	Numeric

C17

		Value
Standard Attributes	Label	Language17
	Type	Numeric

C18

		Value
Standard Attributes	Label	Language18
	Type	Numeric

C19

		Value
Standard Attributes	Label	Language19
	Type	Numeric

C20

		Value
Standard Attributes	Label	Language20
	Type	Numeric

C21

		Value
Standard Attributes	Label	Language21
	Type	Numeric

C22

		Value
Standard Attributes	Label	Language22
	Type	Numeric

C23

		Value
Standard Attributes	Label	Language23
	Type	Numeric

C24

		Value
Standard Attributes	Label	Language24
	Type	Numeric

C25

		Value
Standard Attributes	Label	Language25
	Type	Numeric

C26

		Value
Standard Attributes	Label	Language26
	Type	Numeric

C27

		Value
Standard Attributes	Label	Language27
	Type	Numeric

C28

		Value
Standard Attributes	Label	Language28
	Type	Numeric

C29

		Value
**Standard Attributes**	**Label**	Language29
	**Type**	**Numeric**

C30

		Value
**Standard Attributes**	**Label**	Language30
	**Type**	**Numeric**

C31

		Value
**Standard Attributes**	**Label**	Language31
	**Type**	**Numeric**

C32

		Value
**Standard Attributes**	**Label**	Language32
	**Type**	**Numeric**

C33

		Value
**Standard Attributes**	**Label**	Language33
	**Type**	**Numeric**

C34

		Value
**Standard Attributes**	**Label**	Language34
	**Type**	**Numeric**

D1

		Value
**Standard Attributes**	**Label**	GrossMotor1
	**Type**	**Numeric**

D2

		Value
**Standard Attributes**	**Label**	GrossMotor2
	**Type**	**Numeric**

D3

		Value
**Standard Attributes**	**Label**	GrossMotor3
	**Type**	**Numeric**

D4

		Value
**Standard Attributes**	**Label**	GrossMotor4
	**Type**	**Numeric**

D5

		Value
**Standard Attributes**	**Label**	GrossMotor5
	**Type**	**Numeric**

D6

		Value
**Standard Attributes**	**Label**	GrossMotor6
	**Type**	**Numeric**

D7

		Value
**Standard Attributes**	**Label**	GrossMotor7
	**Type**	**Numeric**

D8

		Value
**Standard Attributes**	**Label**	GrossMotor8
	**Type**	**Numeric**

D9

		Value
**Standard Attributes**	**Label**	GrossMotor9
	**Type**	**Numeric**

D10

		Value
**Standard Attributes**	**Label**	GrossMotor10
	**Type**	**Numeric**

D11

		Value
**Standard Attributes**	**Label**	GrossMotor11
	**Type**	**Numeric**

D12

		Value
**Standard Attributes**	**Label**	GrossMotor12
	**Type**	**Numeric**

D13

		Value
**Standard Attributes**	**Label**	GrossMotor13
	**Type**	**Numeric**

D14

		Value
Standard Attributes	Label	GrossMotor14
	Type	Numeric

D15

		Value
Standard Attributes	Label	GrossMotor15
	Type	Numeric

D16

		Value
Standard Attributes	Label	GrossMotor16
	Type	Numeric

D17

		Value
Standard Attributes	Label	GrossMotor17
	Type	Numeric

D18

		Value
Standard Attributes	Label	GrossMotor18
	Type	Numeric

D19

		Value
Standard Attributes	Label	GrossMotor19
	Type	Numeric

D20

		Value
Standard Attributes	Label	GrossMotor20
	Type	Numeric

D21

		Value
Standard Attributes	Label	GrossMotor21
	Type	Numeric

D22

		Value
Standard Attributes	Label	GrossMotor22
	Type	Numeric

D23

		Value
Standard Attributes	Label	GrossMotor23
	Type	Numeric

D24

		Value
Standard Attributes	Label	GrossMotor24
	Type	Numeric

D25

		Value
Standard Attributes	Label	GrossMotor25
	Type	Numeric

D26

		Value
Standard Attributes	Label	GrossMotor26
	Type	Numeric

D27

		Value
Standard Attributes	Label	GrossMotor27
	Type	Numeric

D28

		Value
Standard Attributes	Label	GrossMotor28
	Type	Numeric

D29

		Value
Standard Attributes	Label	GrossMotor29
	Type	Numeric

D30

		Value
Standard Attributes	Label	GrossMotor30
	Type	Numeric

D31

		Value
Standard Attributes	Label	GrossMotor31
	Type	Numeric

D32

		Value
Standard Attributes	Label	GrossMotor32
	Type	Numeric

D33

		Value
Standard Attributes	Label	GrossMotor33
	Type	Numeric

D34

		Value
Standard Attributes	Label	GrossMotor34
	Type	Numeric

E1

		Value
Standard Attributes	Label	Does the child go to daycare?
	Type	Numeric

E2

		Value
Standard Attributes	Label	How many chindren's book does the child have?
	Type	Numeric

BooksYesNo

		Value
Standard Attributes	Label	<none>
	Type	Numeric

E3

		Value
**Standard Attributes**	**Label**	Does anyone read to the child?
	**Type**	**Numeric**

E4

		Value
**Standard Attributes**	**Label**	Does anyone tell stories to the child?
	**Type**	**Numeric**

E5

		Value
**Standard Attributes**	**Label**	Does anyone sing songs to the child?
	**Type**	**Numeric**

E6

		Value
**Standard Attributes**	**Label**	Does anyone take the child for a ...
	**Type**	**Numeric**

E7

		Value
**Standard Attributes**	**Label**	Does anyone play with the child?
	**Type**	**Numeric**

E8

		Value
**Standard Attributes**	**Label**	Does anyone names objects, count, or draw with the child?
	**Type**	**Numeric**

E9

		Value
**Standard Attributes**	**Label**	How many toys does the child ...
	**Type**	**Numeric**

E10.1

		Value
**Standard Attributes**	**Label**	Caregiver: Mother
	**Type**	**Numeric**

E10.2

		Value
**Standard Attributes**	**Label**	Caregiver: Father
	**Type**	**Numeric**

E10.3

		Value
**Standard Attributes**	**Label**	Caregiver: Stepmother
	**Type**	**Numeric**

E10.4

		Value
**Standard Attributes**	**Label**	Caregiver: Stepfather
	**Type**	**Numeric**

E10.5

		Value
**Standard Attributes**	**Label**	Caregiver: Sister
	**Type**	**Numeric**

E10.6

		Value
**Standard Attributes**	**Label**	Caregiver: Brother
	**Type**	**Numeric**

E10.7

		Value
**Standard Attributes**	**Label**	Caregiver: Grandmother
	**Type**	**Numeric**

E10.8

		Value
**Standard Attributes**	**Label**	Caregiver: Grandfather
	**Type**	**Numeric**

E10.9

		Value
**Standard Attributes**	**Label**	Caregiver: Cousin – female
	**Type**	**Numeric**

E10.10

		Value
**Standard Attributes**	**Label**	Caregiver: Cousin – male
	**Type**	**Numeric**

E10.11

		Value
**Standard Attributes**	**Label**	Caregiver: Other
	**Type**	**Numeric**

E10.12

		Value
**Standard Attributes**	**Label**	Caregiver: Mother and Father
	**Type**	**Numeric**

E10.13

		Value
**Standard Attributes**	**Label**	Caregiver: Mother and Grandmother
	**Type**	**Numeric**

E10.14

		Value
**Standard Attributes**	**Label**	Caregiver: Brother and sister
	**Type**	**Numeric**

E10.15

		Value
**Standard Attributes**	**Label**	Caregiver: Mother and other
	**Type**	**Numeric**

E10.16

		Value
**Standard Attributes**	**Label**	Caregiver: Blank
	**Type**	**String**

E11

		Value
**Standard Attributes**	**Label**	Did the child have low birth weight when he/she was born?
	**Type**	**Numeric**

E12

		Value
**Standard Attributes**	**Label**	Was the child premature?
	**Type**	**Numeric**

E13

		Value
**Standard Attributes**	**Label**	Mother education
	**Type**	**Numeric**
**Valid Values**	**1**	**Preeschool**
	**2**	**Elementary**
	**3**	**Secondary**
	**4**	Undergraduate
	**5**	Post graduate
	**6**	**Master's**
	**7**	**Doctorate**
	**8**	Does not know

TotalA

		Value
**Standard Attributes**	**Label**	Sub-scale score Social
	**Type**	**Numeric**

TotalB

		Value
**Standard Attributes**	**Label**	Sub-scale score Fine motor
	**Type**	**Numeric**

TotalC

		Value
**Standard Attributes**	**Label**	Sub-scale score Language
	**Type**	**Numeric**

TotalD

		Value
**Standard Attributes**	**Label**	Sub-scale score Gross motor
	**Type**	**Numeric**
